# The Importance of Identifying Risk Factors in Childhood and Adolescence

**DOI:** 10.5935/abc.20190016

**Published:** 2019-02

**Authors:** Ana Paula Marte Chacra

**Affiliations:** Instituto do Coração do Hospital das Clínicas da Faculdade de Medicina da Universidade de São Paulo, São Paulo, SP - Brazil

**Keywords:** Dyslipidemias, Obesity, Overweight, Adolescent, Cholesterol LDL-C, Triglycerides, Risk Factors, Atherosclerosis

The study "Dyslipidemia in Adolescents Seen in a University Hospital in the city of Rio de Janeiro/Brazil: Prevalence and Association" showed a high prevalence of obesity (53%) followed by overweight (25.2%) in adolescents. The obese group had a predominance of low HDL-c besides the positive association of body mass index (BMI) and abdominal circumference with triglyceride values.^1^ These date are fundamental and warn of the importance of early assessment of risk factors.

Elevated triglycerides and low HDL-c are strongly linked to obesity, especially in youth^2^ and early exposure to that unfavourable metabolic profile will contribute to a higher cardiovascular risk.^3^

Evidences have shown that atherosclerosis begins in childhood and it is associated with early presence of established risk factors for cardiovascular disease. The progression of atherosclerotic process depends on the time of exposure beyond the interaction between conventional, genetics and environmental risk factors.^4,5^

Despite the early onset of atherogenesis, children and adolescents do not develop clinic manifestations of coronary heart disease, since cardiovascular outcomes depend on prolonged exposure to risk factors. Even so, few longitudinal studies have linked childhood risk factors to adult cardiovascular disease.

Twig et al.^6^ demonstrated association between higher BMI during adolescence with increased cardiovascular mortality in adulthood throughout 40 years of follow-up.^6^ Increased in BMI and triglyceride level was predictive of cardiovascular event in young adulthood, whereas LDL-c levels did not.^7^

Measurements of carotid intima-media thickness (cIMT) by non-invasive imaging techniques provide a surrogate endpoint to assess early atherosclerosis.^8^ Studies have shown that childhood clustering of risk factors are predictive of adult cIMT.^9^

In the study “International Childhood Cardiovascular Cohort (i3C)", Koskinen et al.^10^ demonstrated that obesity, hypertension, and dyslipidemia were predictors of high cIMT in adults.^10^ They found that obesity in children was the most prevalent risk factor associated with high cIMT in adult, increasing the risk by 3.7 times.^10^ Using risk prediction models, when it added the lipid profile to obesity and hypertension, there was a modest improvement in the risk discrimination for increased cIMT in adulthood (area under the curve increased from 0.698-0.717). It may be due to a weak relationship between LDL-c levels and obesity since obesity interferes minimally with LDL-c levels^10^ except where obesity-related metabolic changes unmask an underlying genetic dyslipidemia. In the present cross-sectional study^1^ obesity seems to be the driver of the lipid changes as prevalence of low HDL-c and association of abdominal adiposity with triglycerides levels, without changes in LDL-c values.^1^ Despite these findings, high LDL-c is a well-established risk factor for atherosclerosis as observed in familial hypercholesterolemia, and early detection allows the initiation of pharmacological therapy even in the children.^11^

The present study reinforces that current obesity is a growing epidemic.^1^ The Universal screening would allow for earlier diagnosis and intervention for children with dyslipidemia secondary to lifestyle or genetic factors.^12^
